# Preliminary results on a proposed histopathological assessment of predictive factors for basal cell carcinoma recurrence after primary free margin excision

**DOI:** 10.1002/ski2.88

**Published:** 2022-03-25

**Authors:** A. Jacquet, V. Dormoy, M. Lorenzato, A. Durlach

**Affiliations:** ^1^ Service of Pathology CHU Reims University Hospital of Reims Reims France; ^2^ Inserm P3Cell UMR‐S1250 SFR CAP‐SANTE University of Reims Champagne‐Ardenne Reims France

## Abstract

**Background:**

Basal cell carcinoma (BCC) incidence is steadily increasing but therapeutic solutions remain limited and present a public health challenge.

**Aims:**

To identify predictive factors of BCC recurrence after primary free margin excision, with automated methods, by evaluating cell proliferation, the Hedgehog pathway activation and primary cilia.

**Materials and Methods:**

This case–control study included 32 patients (16 with recurrence occurring at least 6 months after complete resection, and 16 without recurrence) who underwent surgery for BCC. Formalin‐fixed paraffin‐embedded cutaneous resections were processed for immunohistochemistry or immunostaining with the following primary antibodies: mouse anti‐MCM6, rabbit anti‐ARL13B and rabbit anti‐GLI1.

**Results:**

BCC recurrence after free margin excision was significantly linked to a higher proliferative index (*p* < 0.001) and a lower cilia count (*p* = 0.041) in the primary lesion. No significant differences were observed regarding cilia length (*p* = 0.39) or GLI1‐positive nuclei.

**Discussion:**

The complex interplay between essential signaling pathways, cell proliferation and cilia requires further experimental investigations in the context of BCC recurrence.

**Conclusion:**

A higher proliferative index evaluated with MCM6 antibody could be a useful prognosis marker of BCC risk of recurrence. The lower cilia count in the primary lesion unveiled novel perspectives to understand BCC recurrence molecular mechanisms.

## Graphical abstract

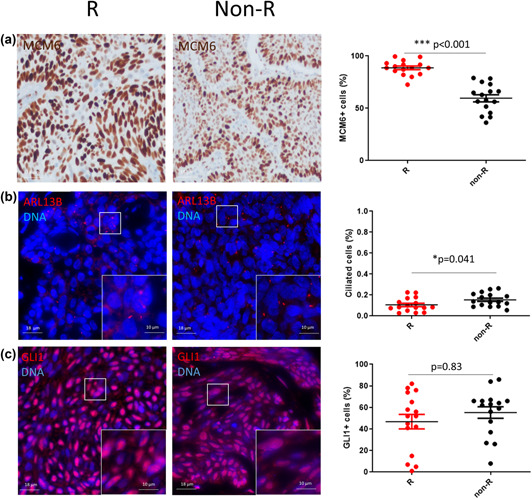

## 1 INTRODUCTION

Basal cell carcinoma (BCC) is the most common skin cancer in Caucasians and the most frequent of all cancers. Its incidence is steadily increasing, but therapeutic solutions remain limited and present a public health challenge.^1^ Recurrence of BCC after primary free margin excision occurs within approximately 2%–10%.^2^

Different proliferative index markers have been extensively studied in BCC, but analyses remained controversial.^3^, ^4^ Mini Chromosome Maintenance proteins (MCM 2–7) are required for cell cycle initiation and DNA replication. They are expressed throughout the cell cycle including cells leaving G0 to enter the early G1 phase, unlike the KI67 assessments.^5^ MCM6 demonstrated its interest in other cancers, such as breast cancer or brain cancer, with a significant clinical association with prognosis or survival in patients.^6^

Recent advances in the understanding of the Hedgehog signalling pathway (specifically GLI1), inherently associated with primary cilia, and its role in BCC pathogenesis led to the introduction of new treatments significantly improving clinical outcomes in unresectable, recurrent or metastatic lesions.^7^ Therefore, cilia identification became an active area of investigation in BCC. ARL13B‐antibody highlights the presence of cilia. This small GTPase operates early in the formation and maintenance of cilia. We hypothesized that ARL13b is a stronger marker of primary cilia to differentiate two BCC that are ciliated. Indeed, most studies analysed primary cilia with acetylated α‐tubulin antibody which underlines cilia presence but also cytoplasmic acetylated cilia. On the contrary, ARL13B is more restrictive: it migrates early during ciliogenesis inside the cilium before acetylation occurs and is found in very low intracytoplasmic levels.^8^

The present study aimed to identify predictive factors of BCC recurrence after primary free margin excision, with automated methods, by evaluating the proliferative activity, the Hedgehog pathway activation and cilia formation.

## 2 REPORT

On a preliminary screen, we selected 281 patients with more than 2 BCC histories (2009–2015 period). Then, we selected 59 patients with two BCC at the same localization as assessed with the dermatology department photo database. When the primary diagnosis was wrong or when margins and histological subtypes were unclear, the patients were rejected. Moreover, one patient refused to be included in our study. Finally, 16 recurrent BCC were selected for analysis, with strict selective criteria. They were matched with non‐recurrent BCC, selected from the 2010–2015 period, and defined as patients with no recurrence during a follow‐up period of at least 5 years and with an average follow‐up of 8.6 years. Microscopic length, deep and lateral margin were analysed. Formalin‐fixed paraffin‐embedded (FFPE) cutaneous resections were processed for immunohistochemistry or immunostaining with the following primary antibodies: mouse anti‐MCM6 (H‐8, Santa Cruz Biotechnology), rabbit anti‐ARL13B (17711–1‐ap, ProteinTech) and rabbit anti‐GLI1 (HPA065172, Sigma Aldrich). Immunoreactivity was illustrated with Ultra View Universal 3,3’‐diaminobenzidine (DAB) detective kit, using the Ventana Automatic Benchmark Ultra System.

For the evaluation of MCM6‐positive cells, five high‐power fields with the highest number of positive nuclei were counted at 400 magnifications.

For the evaluation of GLI1 and cilia, five high‐power fields at 1000 magnifications were processed for quantification.

Micrographs were acquired by AxioImageur Zeiss (100x Ph) with ZEN software. Data were obtained and processed with the ImageJ software. The percentage of ciliated cells and the average cilia length were automatically quantified with CiliaQ on ImageJ.^9^

Comparisons between groups were performed using Student's tests or Wilcoxon–Mann–Witney tests when appropriate.

**FIGURE 1:**
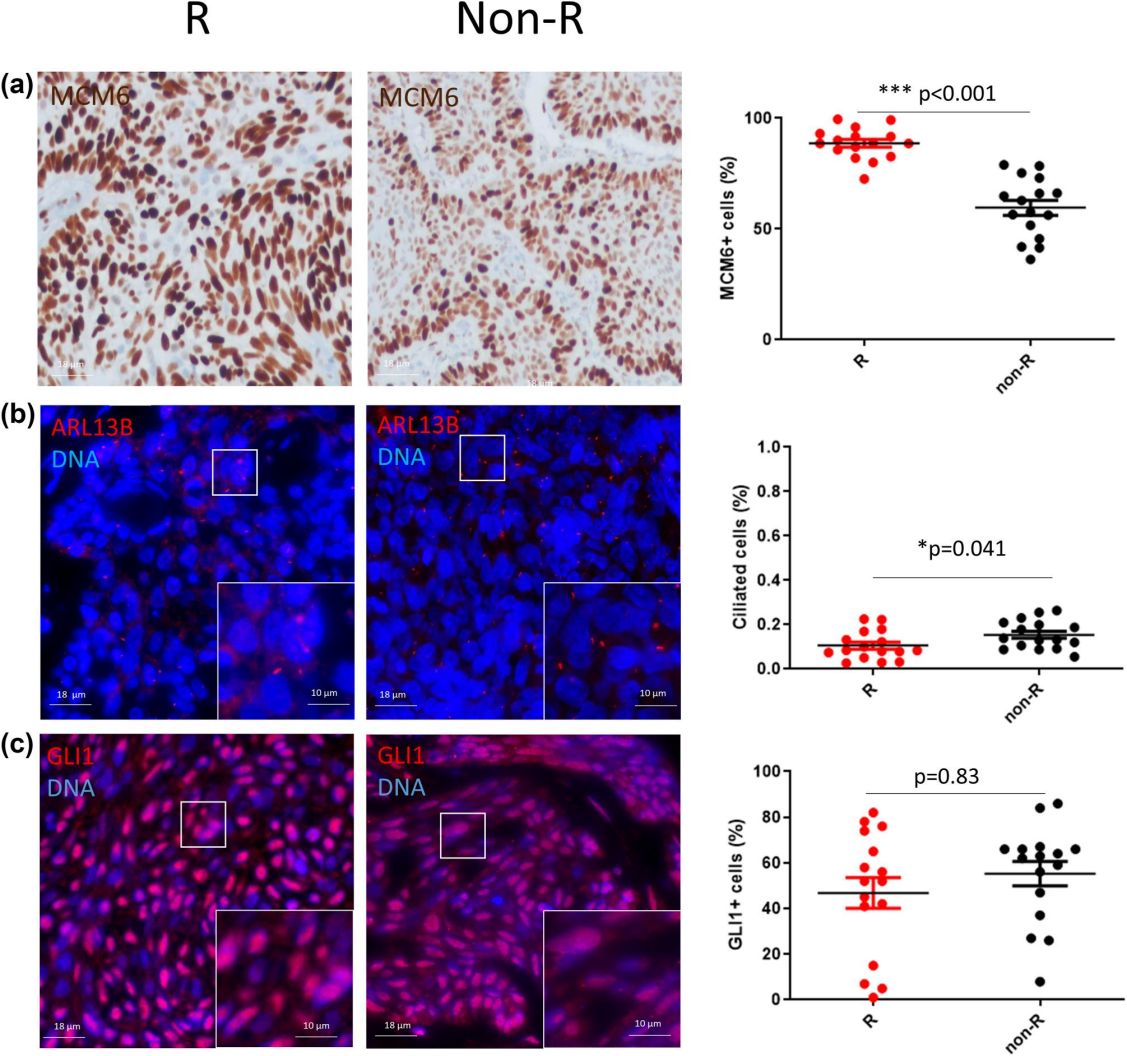
Quantitative assessment of proliferation, Hedgehog pathway activation and cilia in recurrent (R) and non‐recurrent (non‐R) basal cell carcinoma. (a) Representative micrographs showing MCM6+ cells (MCM6, brown; nuclei are counterstained with haematoxylin) and dot plot with mean and SD. (b) Magnification corresponding to the selected area is shown Representative micrographs showing ciliated cells (ARL13B, red; nuclei are stained in blue with DAPI). Magnification corresponding to the selected area is shown. *p < 0.05 and ***p < 0.001; non‐R versus R. (c) Representative micrographs showing GLI1+ cells (GLI1, red; nuclei are stained in blue with DAPI) and dot plot with mean and SD

No significant differences were observed between the two groups, regarding clinical and histological characteristics (Table 1), except for the size of the lesions in the group of primary tumours that eventually recurred (5.75 +/−0.7 mm vs. 7.6 +/−3.6 mm; *p* = 0.05). We found a statistically significant increased proliferative index evaluated with MCM6 (Figure 1a; 88.60 +/−7.04% vs. 59.59 +/−13.52%; *p* < 0.001); and a lower percentage of ciliated cells (Figure 1b; 11 +/−6.4% vs. 15 +/−6%; *p* = 0.041) in recurrent BCC. However, no significant differences were observed regarding cilia length (0.62 +/− 0.17 μm vs. 0.73 +/−0.47 μm, *p* = 0.39) or GLI1‐positive nuclei (Figure 1c; 53 +/−22% vs. 55 +/−21%; *p* = 0.83).

**TABLE 1:** Clinical and histological characteristics of BCC

	Recurrent BCC (*n* = 16)	Non‐recurrent BCC (*n* = 16)	*p*‐value
Age (mean, year) +/‒SD	71.2 +/−11.02	65.29 +/−11.2	0.14
Sex ratio M/F	8/8	8/8	
Head and neck/Trunk localization	7/1	7/1	
Histological subtypes			
Nodular	6	6	
Superficial	1	1	
Infiltrative	8	8	
Basosquamous	1	1	
Tumour length (mm, mean) +/‒SD	5.75 +/−0.71	7.56 +/−3.64	0.05
Lateral margin (mm)			0.11
<1	7	3	
1‒3	9	12	
>3	0	1	
Deep margin (mm)	1.47 +/−0.84	1.82 +/−1.28	0.35
Ulceration	10	7	0.31
TILs			0.41
Absent	7	9	
Brisk	8	7	
Non‐brisk	1	0	

*Note*: Brisk TILs are present throughout the vertical growth phase or across the entire base of the tumour; non‐brisk TILs focally infiltrate the tumour.

Abbreviations: BCC, basal cell carcinoma; TILs, tumour‐infiltrating lymphocytes.

A subgroup analysis was performed based on the classification of the recurrence risk of BCC (seven low‐risk BCC and nine high‐risk BCC). The results were identical and showed a statistically significant association between recurrence and cell proliferation and the decrease in the number of cilia (except for low‐risk BCC). The results remained inconclusive for GLI1.

## 3 DISCUSSION

In this study, we demonstrated that recurrence of BCC was associated with a higher MCM6 proliferative index, with relative slightly dispersed values. This was an important observation since KI67 assessments were often inconclusive in BCC immunohistochemistry analyses because of a large dispersion of the results. This was further associated with a decrease in ciliated cells.

The originality of our approach lies in the evaluation of an additional proliferation‐associated marker (MCM6‐antibody), and in the automated computer‐assisted evaluation of cilia on FFPE tissue sections with ARL13B staining instead of acetylated‐tubulin which provides a more robust evaluation of ciliated cells.^10^ Molecular mechanisms driving the development of BCC are not completely elucidated. Here we highlighted that recurrence was not specifically associated with Hedgehog signalling reactivation in BCC. It was previously reported that BCC recurrence could be linked to the activation of other signalling pathways, intimately linked with Hedgehog pathway and cilia formation, such as Wnt, NOTCH or EGFR and that a cross‐talk existed between these signalling pathway.^11^ It will be interesting to explore these complex cross‐talks in the light of our preliminary findings in future analyses. The evaluation of GLI1 intensity profile with a semi‐quantitative scale may bring additional information. However, due to the high variability of the immunostainings within the same series of specimen, the sample size should be enlarge to apply this method. To consolidate our analysis, additional experimental investigations are required to increase the number of patients, the localizations of BCC, and the quantitative parameters.

In conclusion, we have shown that a higher proliferative index evaluated with MCM6 antibody could be a useful prognosis marker of BCC risk of recurrence. The lower cilia count in the primary lesion (evaluated with ARL13B) unveiled novel perspectives to understand BCC recurrence molecular mechanisms.

## CONFLICT OF INTEREST

Valérian Dormoy reports personal fees from Chiesi outside the submitted work.

## AUTHOR CONTRIBUTIONS

**A. Jacquet:** Conceptualization, Data curation, Formal analysis, Funding acquisition, Investigation, Methodology, Project administration, Resources, Software, Supervision, Validation, Visualization, Writing – original draft, Writing – review & editing. **V. Dormoy:** Conceptualization, Data curation, Formal analysis Funding acquisition, Investigation, Methodology, Project administration, Resources, Software, Supervision, Validation, Visualization, Writing – original draft, Writing – review & editing. **M. Lorenzato:** Conceptualization, Data curation, Formal analysis, Methodology, Supervision, Writing – original draft, Writing – review & editing. **A. Durlach:** Conceptualization, Data curation, Formal analysis, Funding acquisition, Investigation, Methodology, Project administration, Resources, Software, Supervision, Validation, Visualization, Writing – original draft, Writing – review & editing.

## Data Availability

The data that support the findings of this study are available on request from the corresponding author. The data are not publicly available due to privacy or ethical restrictions.
